# Heme and non-heme iron transporters in non-polarized and polarized cells

**DOI:** 10.1186/1471-2121-11-39

**Published:** 2010-06-04

**Authors:** Izumi Yanatori, Mitsuaki Tabuchi, Yasuhiro Kawai, Yumiko Yasui, Reiko Akagi, Fumio Kishi

**Affiliations:** 1 Department of Molecular Genetics, Kawasaki Medical School, Okayama 701-0192, Japan; 2 Department of Pharmacy, Faculty of Pharmacy, Yasuda Women's University, Hiroshima 731-0153, Japan

## Abstract

**Background:**

Heme and non-heme iron from diet, and recycled iron from hemoglobin are important products of the synthesis of iron-containing molecules. In excess, iron is potentially toxic because it can produce reactive oxygen species through the Fenton reaction. Humans can absorb, transport, store, and recycle iron without an excretory system to remove excess iron. Two candidate heme transporters and two iron transporters have been reported thus far. Heme incorporated into cells is degraded by heme oxygenases (HOs), and the iron product is reutilized by the body. To specify the processes of heme uptake and degradation, and the reutilization of iron, we determined the subcellular localizations of these transporters and HOs.

**Results:**

In this study, we analyzed the subcellular localizations of 2 isoenzymes of HOs, 4 isoforms of divalent metal transporter 1 (DMT1), and 2 candidate heme transporters--heme carrier protein 1 (HCP1) and heme responsive gene-1 (HRG-1)--in non-polarized and polarized cells. In non-polarized cells, HCP1, HRG-1, and DMT1A-I are located in the plasma membrane. In polarized cells, they show distinct localizations: HCP1 and DMT1A-I are located in the apical membrane, whereas HRG-1 is located in the basolateral membrane and lysosome. 16Leu at DMT1A-I N-terminal cytosolic domain was found to be crucial for plasma membrane localization. HOs are located in smooth endoplasmic reticulum and colocalize with NADPH-cytochrome P450 reductase.

**Conclusions:**

HCP1 and DMT1A-I are localized to the apical membrane, and HRG-1 to the basolateral membrane and lysosome. These findings suggest that HCP1 and DMT1A-I have functions in the uptake of dietary heme and non-heme iron. HRG-1 can transport endocytosed heme from the lysosome into the cytosol. These localization studies support a model in which cytosolic heme can be degraded by HOs, and the resulting iron is exported into tissue fluids via the iron transporter ferroportin 1, which is expressed in the basolateral membrane in enterocytes or in the plasma membrane in macrophages. The liberated iron is transported by transferrin and reutilized for hemoglobin synthesis in the erythroid system.

## Background

Iron has an essential function in mammalian metabolism because of the ease with which it can gain and lose electrons. It is involved in biological functions as a metal cofactor for many proteins and enzymes that are used in oxygen transport (hemoglobin and myoglobin), electron transfer (mitochondrial cytochrome), and DNA synthesis (ribonucleotide reductase). Thus, iron is indispensable for eukaryotes and prokaryotes; however, it is also potentially toxic because of the generation of the superoxide anion and hydroxyl radical. These oxygen metabolites readily react with biological molecules, including proteins, lipids, and DNA. Iron overload diseases owing to genetic misregulation of iron uptake are referred to as primary iron overload disease or hereditary hemochromatosis [1]. On the other hand, an acquired anemia that is associated with iron deficiency is referred to as anemia of inflammation or anemia of chronic disease [2]. Organisms have a system to maintain normal iron homeostasis; iron deficiency and overload are associated with cellular dysfunction. Therefore, all mammalian species tightly regulate the iron concentration in body fluids. Because humans lack a regulated pathway for iron excretion, regulation of iron absorption from the intestine and the recycling of iron from senescent red blood cells (RBCs) are crucial in maintaining iron balance. Normal iron loss in humans occurs through exfoliation of enterocytes and skin cells, and through menstruation and childbirth. The absorption of dietary iron, composed mostly of heme and non-heme iron, occurs predominantly in the duodenum and upper jejunum, and is highly regulated [3]; this involves transport of absorptive enterocytes across the apical membrane into the cytosol and across the basolateral membrane into body fluids. Divalent metal transporter 1 (DMT1) is the only known intestinal iron importer and is a member of the natural resistance-associated macrophage protein family [4-7]. DMT1 is highly conserved from prokaryotes to eukaryotes, expressed in the apical membrane of absorptive enterocytes in the small intestine, and is also present in the endosomes of all human cells [8]. Proper endosomal recycling of DMT1 is important for efficient uptake of iron and depends on a retromer-mediated sorting mechanism [9]. Iron imported by DMT1 enters into the cytosol of the absorptive cells where it can be stored in the cytosolic iron-storage molecule ferritin or exported into body fluids through the basolateral iron exporter ferroportin 1 (FPN1) [10-12]. FPN1, the only known cellular iron exporter, is found on all cell types, including the duodenal mucosa, macrophage, and placenta. The bactericidal peptide hepcidin functions as an iron regulatory hormone [13,14]. The hepcidin gene encodes an 84-amino-acid pre-pro-peptide that is cleaved to form a bioactive 25-amino-acid peptide found in the plasma and urine. Hepcidin is synthesized in the liver, and its gene expression is increased by iron overload and inflammation, especially interleukin 6 and interleukin 1 [15], and decreased by hypoxia and anemia [16]. Hepcidin induces irreversible internalization of FPN1 through lysosomal degradation, which results in a depletion of plasma iron and an accumulation of iron in duodenal enterocytes and macrophages [13].

Humans are able to utilize 2 types of iron, heme and non-heme. Heme is an important nutritional source of iron and is believed to be more readily absorbed than non-heme iron. Heme is a ubiquitous molecule with an active iron center carrying a high affinity to oxygen, which allows for reversible binding and transport of oxygen in hemoglobin. Heme groups serve as the catalytic site; they tightly bind to a variety of proteins involved in aerobic metabolism, including respiratory chain cytochromes and numerous cytochrome P450 isoenzymes. Heme is mostly absorbed in the proximal half of the duodenum, the absorptive capacity of which is decreased in the distal position of the small intestine [17]. In macrophage, senescent RBCs are phagocytosed and digested into heme in the lysosome. Heme degradation is catalyzed by heme oxygenases (HOs), the activities of which are particularly high in the spleen, testes, brain, and liver [18]. At present, cDNAs encoding 2 isoenzymes, HO-1 [19] and HO-2 [20], have been cloned. Although HO-1 and HO-2 catalyze the same reaction and have similar cofactor requirements (NADPH-cytochrome P450 reductase and O_2_) [21], they substantially differ in regulation and expression patterns. HO-1 and HO-2 proteins differ in molecular weight. HO-1 is an inducible isoenzyme, while HO-2 is constitutive. HO-1 has been identified as the major 32-kDa heat shock protein hsp32 [19] and is highly sensitive to various stimuli, including oxidative stress, heavy metals, UV radiation, and inflammation. Several reports investigated HO localization to various subcellular compartments, including endoplasmic reticulum (ER) [22], nucleus [23], mitochondria [24], or caveola [25]. HO-1 has also been reported to change its location under hemin treatment [23,26].

Because the catalytic sites of HOs are supposed to be in the cytosol, heme needs appropriate transporters for its import into the cytosol through the plasma or endosomal membrane. Two heme transporters have thus far been reported--heme carrier protein 1 (HCP1) [27] and heme responsive gene-1 (HRG-1) [28]. HCP1 is highly conserved and is a member of a large family of proton-coupled transporters known as the major facilitator superfamily. Within this family, HCP1 well resembles a bacterial protein that transports the antibiotic tetracycline [27]. Notably, there are structural similarities between the planar heme ring and tetracycline-metal structures that must be transported across the apical membrane of absorptive enterocytes. Moreover, HCP1 has recently been identified as a transporter that mediates the translocation of folate across the plasma membrane and is suggested to be the possible molecular entity of the carrier-mediated intestinal folate transport system [29]. HRG-1 and HRG-4 have been reported to be essential in heme homeostasis and heme sensing in *Caenorhabditis elegans*, and HRG-1 knockdown leads to profound defects in erythropoiesis in zebrafish [28]. HCP1 was found to be expressed in the duodenum and small intestine [27], and HRG-1 in the brain, heart, kidney, and small intestine [28]. The expression of HCP1 and HRG-1 was also investigated in some cultured cell lines and detected in macrophage and epithelial cell lines. It is not clear yet which transporter predominantly functions as the heme transporter in enterocytes or macrophages, both of which are main iron-regulatory cells in the human body.

To understand heme catabolism in humans, it is important to analyze the relationship among heme transporters, HOs, and iron transporters. In the current study, we investigate the expression and subcellular localization of HOs, DMT1, HRG-1, and HCP1 in non-polarized and polarized epithelial cells. Our results suggest that HCP1 functions on the apical membrane of enterocytes, HRG-1 transports heme from the inside of the lysosome into the macrophages, and HOs on smooth ER catalyze the degradation of heme in the cytosol.

## Results

### HOs are localized to smooth ER

**Figure 1:**
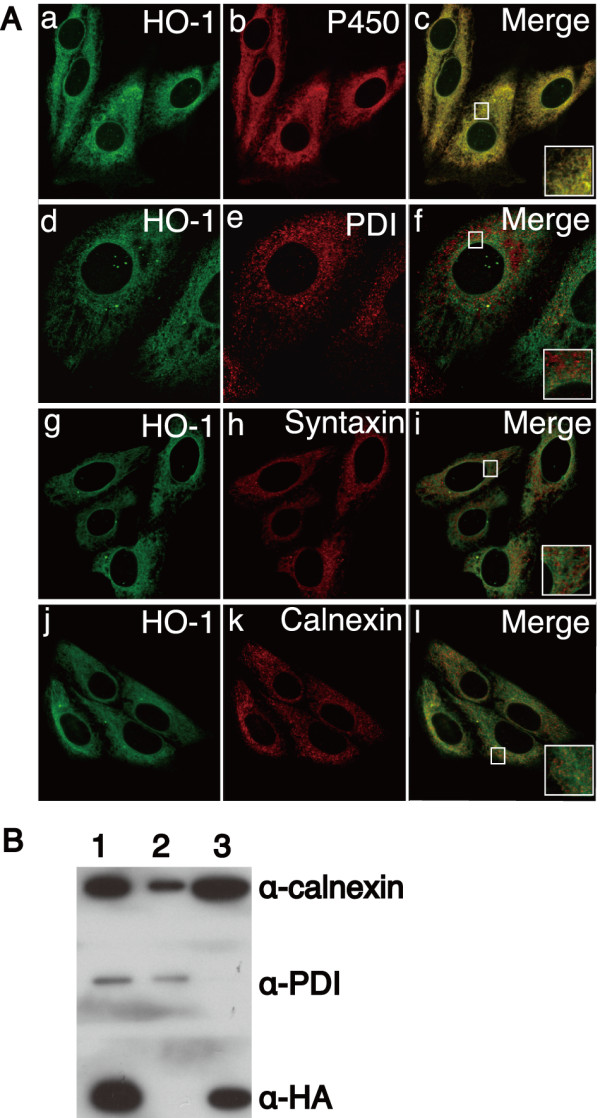
**HOs are localized to smooth ER**. A. GFP-tagged HO-1 was cotransfected with mCherry-tagged NADPH-cytochrome P450 reductase or mCherry-tagged syntaxin 17 into HEp-2 cells and visualized by confocal microscopy. Cells were fixed and incubated with antibodies against GFP (a, d, g, and j), mCherry (b and h), PDI (e), and calnexin (k). Each inset shows a higher magnification image of the boxed area. B. Separation of rough and smooth microsomes. The subcellular fractionation method is described in detail in the Methods section. Proteins were detected by anti-HA, anti-PDI, and anti-calnexin mAbs. Lane 1, post mitochondrial supernatant; lane 2, rough microsomal fraction; lane 3, smooth microsomal fraction. Similar results were obtained in 3 independent experiments.

Four types of HO-1 and HO-2 constructs were constructed to examine the effect of the tagging molecule, GFP or HA, on the localization of HOs (Additional file 1 Figure. S1B). HO localization was not affected by the addition of either of these two tags on either the N or C terminus. HO-1 and HO-2 are completely colocalized with each other (Additional file 1 Figure. S1C). Thus, we used HOs with a suitable tag for each experiment throughout this study. Previous reports show that HO-1 is localized to the endosome or caveola. We compared the localization of HO-1 with some endosome markers or caveola. GFP-tagged HO-1 was not colocalized with TfR (recycling endosome), LAMP2 (late endosome/lysosome), EEA1 (early endosome), or caveolin (Additional file 2 Figure. S2). GFP-tagged HO-1 was cotransfected with mCherry-tagged NADPH-cytochrome P450 reductase [32] or mCherry-tagged syntaxin 17 [33] (Figure. 1A). Cells were additionally immunostained with anti-calnexin or anti-PDI mAb. NADPH-cytochrome P450 reductase is a donor protein that gives an electron to HOs. NADPH-cytochrome P450 reductase is speculated to be located in smooth ER [32]. Syntaxin 17 and calnexin are located in both rough and smooth ER [33,34]. PDI catalyses disulfide interchange between thiols and protein dilsulfides, and has the highly conserved ER retention sequence Lys-Asp-Glu-Leu (KDEL) in its C terminus [35,36]. GFP-tagged HO-1 completely colocalized with NADPH-cytochrome P450 reductase (Figure. 1A, a-c) and partly colocalized with syntaxin 17 (Figure. 1A, g-i) and calnexin (Figure. 1A, j-k). On the other hand, GFP-tagged HO-1 showed distinct localization from that of PDI, which is located in rough ER (Figure. 1A, d-f). To separate rough and smooth microsomes, subcellular fractionation was performed with a 2-layered sucrose gradient using HEp-2 cells stably expressing HA-tagged HO-2. In the rough microsomal fraction, we did not observe HO-2 but observed both calnexin and PDI (Figure. 1B, lane 2). In the smooth microsomal fraction, mainly HO-2 and calnexin were detected (Figure. 1B, lane 3). These data strongly support the results obtained by immunofluorescence microscopic analysis. Hence, it is assumed that HOs are located on the smooth ER membrane and not on the rough ER membrane.

### HO-1 does not change its location under hemin treatment

**Figure 2:**
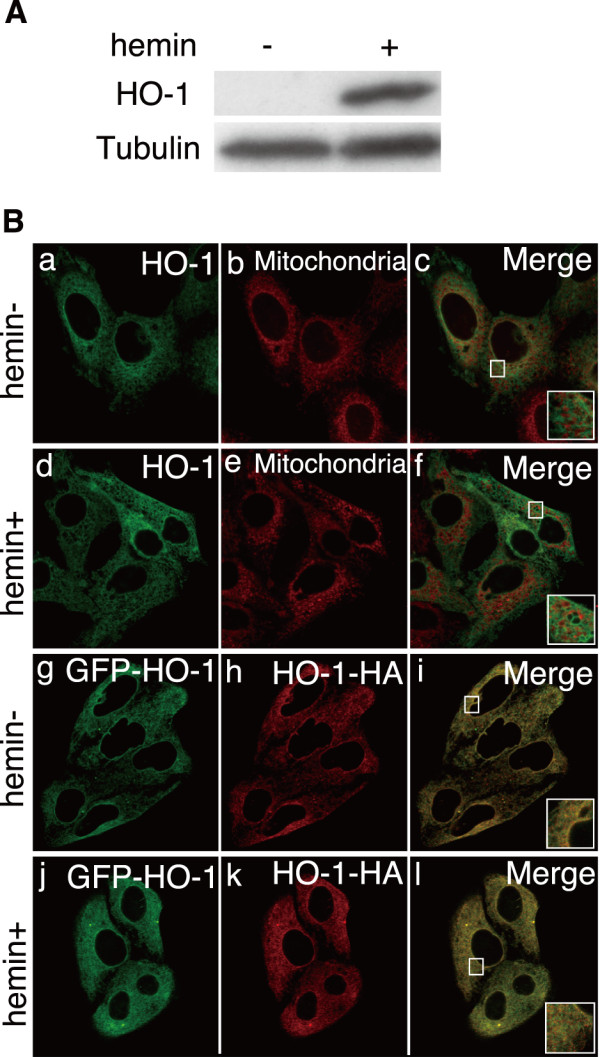
**HO-1 localization is not changed under the hemin treatment**. A. Western blot analysis of endogenous HO-1. HEp-2 cells were treated with 100 μM hemin and cultured for 36 h. Tubulin was used as an internal control. B. N-terminally GFP-tagged HO-1 was transfected into HEp-2 cells (a-f) and cultured for 36 h with 100 μM hemin. Cells were incubated with 250 nM MitoTracker Deep Red (b and e) for 45 min, and then fixed with 4% PFA. N-terminally GFP-tagged HO-1 and C-terminally HA-tagged HO-1 were cotransfected into HEp-2 cells and incubated with 100 μM hemin for 36 h. The cells were fixed and stained with antibodies against GFP (g and j) and HA (h and k). Each inset shows a higher magnification image of the boxed area.

HO-1 is highly inducible by hemin and is thought to inhibit inflammation and protect against oxidative damage [37]. Before the examination of HO localization under hemin treatment, hemin was confirmed to induce gene expression of endogenous HO-1 in HEp-2 cells (Figure. 2A). Previous reports show that under hemin treatment, HO-1 was cut off from its C-terminal transmembrane region and then relocated to the nucleus [23] or mitochondria [26]. If the protease that cuts off the C-terminal half of HO-1 is induced by hemin, both endogenous and transfected HO-1 should be modified by this protease, and the change of localization should be observed in both HO-1 molecules. Endogenous HO-1 molecule could not be detected without hemin induction (Figure. 2A) and it was impossible to compare the localization of endogenous HO-1 with or without hemin treatment. Then, we analyzed the possible change of HO-1 localization by using recombinant HO-1 molecule. N-terminally GFP-tagged HO-1 was transfected into HEp-2 cells, and then cells were cultured with 100 μM hemin for 36 h. Transfected GFP-tagged HO-1 did not change its location to the nucleus or mitochondria (Figure. 2B, a-f); this suggests that the localization of the N-terminal half of HO-1 is not affected by hemin. Because it can be presumed that under hemin treatment, the C-terminal half of HO-1 changes its location to the nucleus or mitochondria after being cut off by the protease, we investigated whether C-terminally HA-tagged HO-1 could change its location under hemin treatment. N-terminally GFP-tagged HO-1 and C-terminally HA-tagged HO-1 were cotransfected and their localizations under hemin treatment were analyzed. N-terminally tagged HO-1 and C-terminally tagged HO-1 still completely colocalized with each other (Figure. 2B, g-l). Therefore, these results indicate that the localization of HO-1 does not switch from ER to the nucleus or mitochondria under hemin treatment.

### HRG-1 and HCP1 are localized to the plasma membrane and lysosome

**Figure 3:**
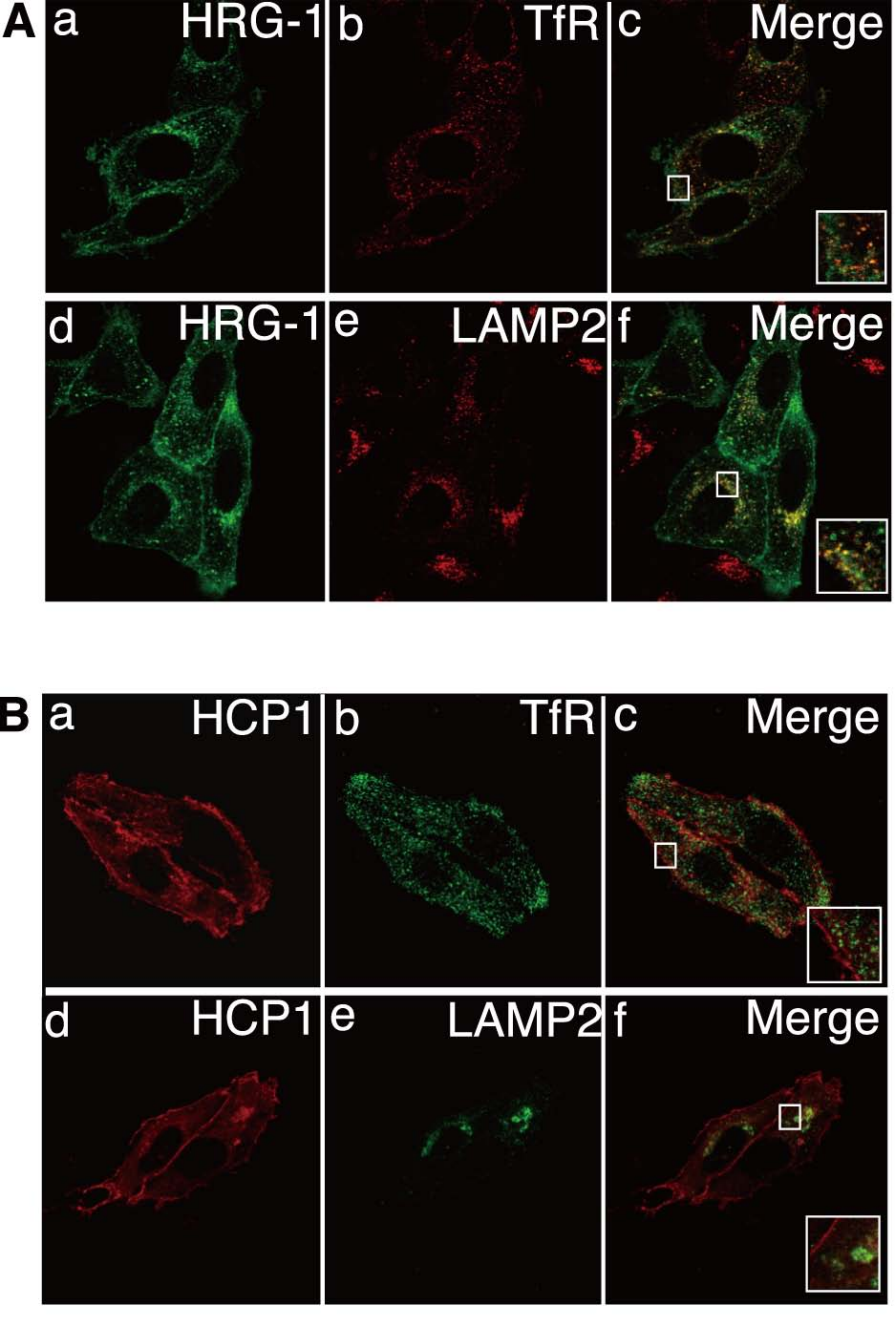
**Subcellular localizations of HRG-1 and HCP1 in HEp-2 cells**. A. GFP-tagged HRG-1 was transfected into HEp-2 cells. The cells were fixed and stained with antibodies against GFP (a and d), TfR (b), and LAMP2 (e). B. mCherry-tagged HCP1 was transfected into HEp-2 cells. The cells were fixed and stained with antibodies against mCherry (a and d) TfR (b), and LAMP2 (e). Each inset shows a higher magnification image of the boxed area.

Two heme transporter candidates have recently been reported: HCP1 and HRG-1. We analyzed their subcellular localizations. HRG-1 was localized to the plasma membrane and partly colocalized with TfR (Figure. 3A, a-c) and LAMP2 (Figure. 3A, d-f). HRG-1 has been reported to be trafficked from the endosome to the plasma membrane when the cells were cultured under serum-starvation conditions [38]. We examined HRG-1 translocation using HEp-2 and MDCK cells stably expressing GFP-tagged HRG-1 under serum starvation. In 2 h (Additional file 3 Figure. S3b, e) or 24 h (Additional file 3 Figure. S3c, f) under serum starvation, we observed HRG-1 both in the cytosolic organelles and plasma membrane. HRG-1 translocation was not observed under these conditions. HCP1 was localized to the plasma membrane and partly colocalized with LAMP2 (Figure. 3B, d-f) but not with TfR (Figure. 3B, a-c). These results suggest that both HRG-1 and HCP1 are located in the plasma and lysosomal membranes in HEp-2 cells.

### Subcellular localizations of 4 isoforms of the iron transporter DMT1

**Figure 4:**
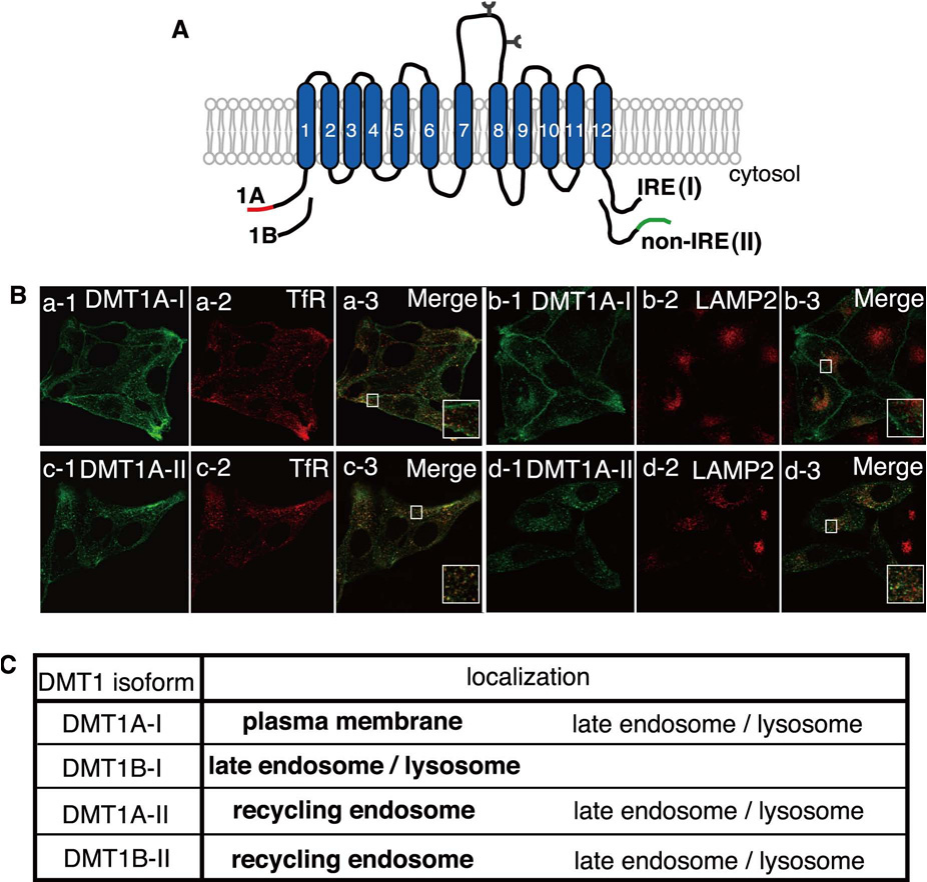
**Subcellular localizations of 4 DMT1 isoforms**. A. Four isoforms of DMT1. B. GFP-tagged DMT1A-I (a and b) or GFP-tagged DMT1A-II (c and d) was transfected into HEp-2 cells. The cells were fixed and stained with antibodies against TfR (a-2 and c-2) and LAMP2 (b-2 and d-2). C. The localizations of the 4 DMT1 isoforms are indicated. Bold letters indicate the main localization of each isoform.

DMT1 has 4 isoforms. The differences in their N-terminal regions are produced by 2 different promoters (1A or 1B in Figure. 4A), whereas those in their C-terminal regions are produced by alternative splicing [IRE (I) or non-IRE (II) in Figure. 4A] [6,8,39,40]. Each of the DMT1 isoforms has been reported to be expressed in specific tissues or cells [40]. Thus, each DMT1 isoform was transfected into HEp-2 cells and compared with several organelle markers. As shown in Figure. 4, DMT1A-I was mainly localized in the plasma membrane and partly colocalized with LAMP2 but not with TfR (Figure. 4B, a-1 to b-3), whereas DMT1A-II colocalized with TfR (Figure. 4B, c-1 to d-3). The localizations of the DMT1B-I and DMT1B-II isoforms were previously reported [40] (data not shown). These results show that the C-terminal region conducts the subcellular localization of DMT1A as in the case of DMT1B, and the addition non-IRE (II) C-terminal cytosolic domain leads DMT1A and DMT1B to the recycling endosome. These data are summarized in Figure. 4C. The main locations of each DMT1 isoform are as follows: DMT1A-I, plasma membrane; DMT1A-II, recycling endosome; DMT1B-I, late endosome and lysosome; DMT1B-II, recycling endosome.

### 16Leu at the N-terminal cytosolic domain is a crucial signal for DMT1A-I plasma membrane localization

**Figure 5:**
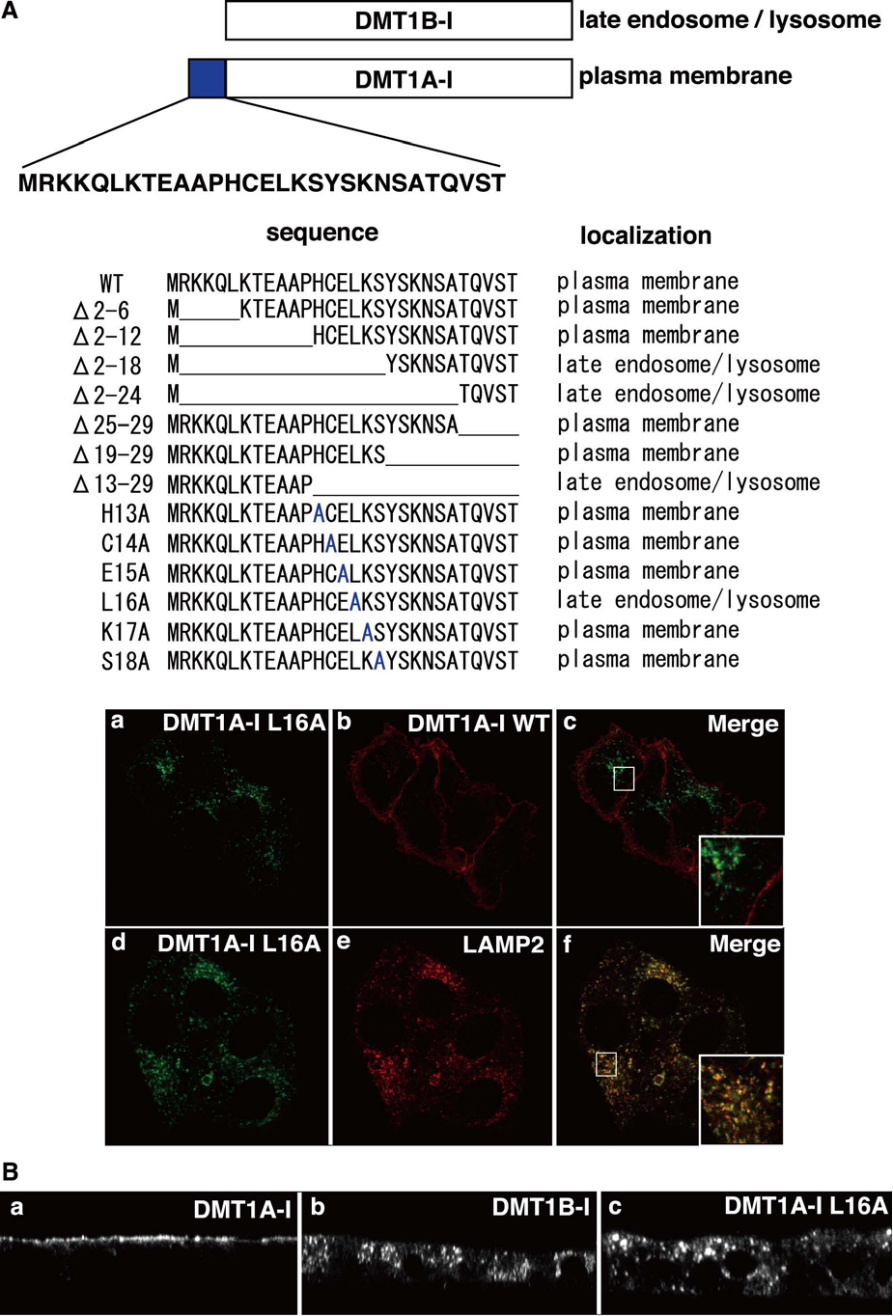
**Mutational analysis of the N-terminal cytoplasmic domain of DMT1A-I reveals the crucial signal for localizing to the plasma membrane**. A. Summary of the mutational analysis of the N-terminal cytoplasmic domain of DMT1A-I. Amino acid sequences of the N-terminal cytoplasmic domain of wild-type DMT1A-I and its various mutants are displayed together with their localizations. GFP-tagged DMT1A-I L16A (a and d) and mCherry-tagged DMT1A-I WT (b) are transfected into HEp-2 cells. The cells were fixed and stained with antibodies against LAMP2 (e). B. MDCK cells stably expressing GFP-tagged DMT1A-I (a), GFP-tagged DMT1B-I (b), and GFP-tagged DMT1A L16A (c) were grown on Transwell and analyzed after their polarization.

To identify the structural requirements for the plasma membrane localization signal of DMT1A-I, we performed a detailed mutational analysis of the N-terminal cytosolic domain sequence. We constructed various mutants of DMT1A-I, which had deletions or amino acid substitutions within the 29-amino-acid sequence specific for DMT1A (Figure. 5A). Deletion analyses showed that the His13-Ser18 region contains the crucial region for DMT1A-I localization to the plasma membrane. Then, we performed alanine scanning to investigate which amino acid in this region is crucial for DMT1A-I localization. Notably, the L16A mutant displayed a severe mislocalization of DMT1A-I to late endosome and lysosome (Figure. 5A, a-f). Then, we constructed MDCK cells stably expressing GFP-tagged DMT1A-I, GFP-tagged DMT1B-I, or GFP-tagged DMT1A-IL16A to investigate the localization in polarized cells. We observed that DMT1A-I was localized to the apical membrane and DMT1B-I to the cytosolic organelle. DMT1A-IL16A was not localized to the apical membrane but was in the cytosolic organelle, as was observed in DMT1B-I.

### HRG-1 localization is distinct from that of HCP1 or DMT1A-I in polarized cells

**Figure 6:**
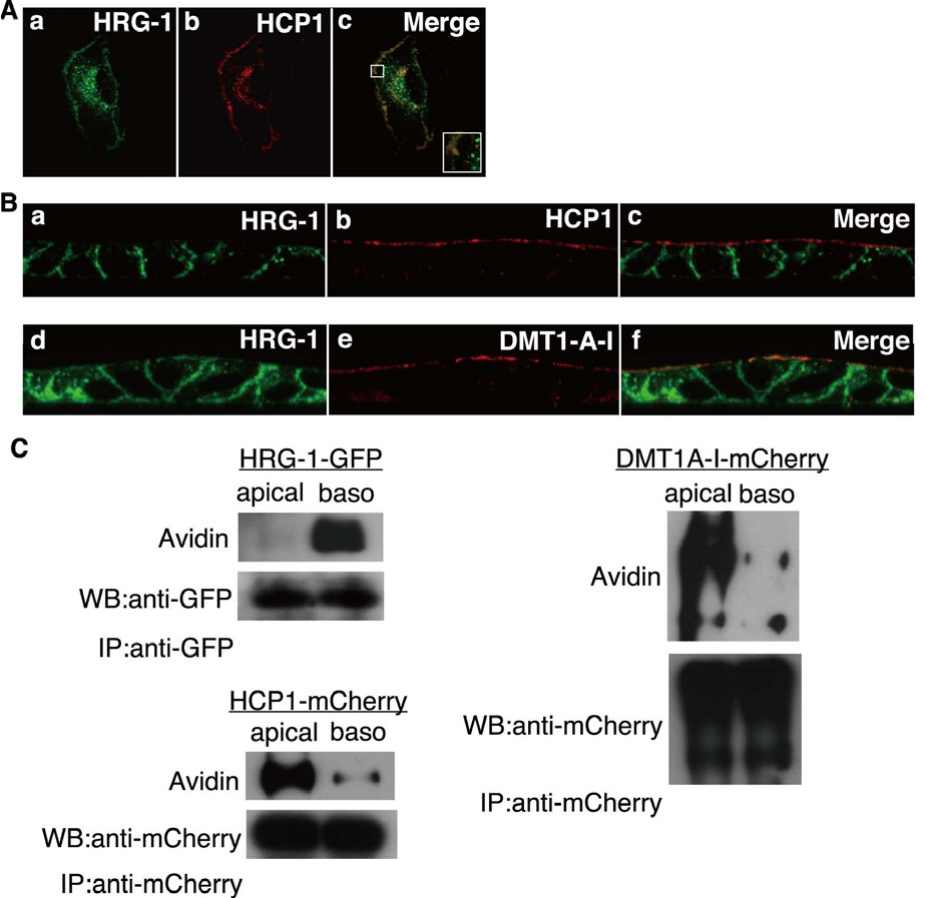
**Subcellular localizations of HRG-1, HCP1, and DMT1A-I in polarized or non-polarized MDCK cells**. A. GFP-tagged HRG-1 (a) or mCherry-tagged HCP1 (b) were cotransfected into HEp-2 cells. The cells were cultured on glass coverslips for 48 h after replating. B. MDCK cells stably expressing GFP-tagged HRG-1 (a and d) and mCherry-tagged HCP1 (b) or mCherry-tagged DMT1A-I (e) were grown on Transwell and analyzed after their polarization. C. Cell surface labeling assay. MDCK cells stably expressing GFP-tagged HRG-1, mCherry-tagged HCP1, or mCherry-tagged DMT1A-I were grown on Transwell, and each apical or basolateral surface proteins were biotinylated. The biotinylated proteins were immunoprecipitated with protein A conjugated with anti-GFP or anti-mCherry polyclonal antibodies, and analyzed by Western blot. Similar results were obtained in 3 independent experiments.

GFP-tagged HRG-1 and mCherry-tagged HCP1 showed very similar localizations in HEp-2 cells (Figure. 6A); we also examined HRG-1, HCP1, and DMT1A-I localizations in polarized MDCK cells (Figure. 6B). GFP-tagged HRG-1 and mCherry-tagged HCP1 were cotransfected in HEp-2 cells. HRG-1 colocalized with HCP1 in the plasma membrane and partly in the cytosolic organelle of non-polarized HEp-2 cells (Figure. 6A). HRG-1 showed much more cytosolic accumulation, and HCP1 showed thick accumulation in the plasma membrane (Figure. 6A, a-c). Then, we constructed MDCK cells stably expressing GFP-tagged HRG-1 and mCherry-tagged HCP1 or mCherry-tagged DMT1A-I to investigate the localization of HRG-1, HCP1, and DMT1A-I in polarized MDCK cells. HRG-1 was detected in the basolateral membrane and in the cytosolic organelle just under the apical membrane (Figure. 6B, a, d). On the other hand, HCP1 (Figure. 6B, b) and DMT1A-I (Figure. 6B, e) were mainly localized to the apical membrane. Figure 3 shows that this cytosolic organelle is assumed to be late endosome and lysosome. To confirm our immunofluorescence assay, the cell surface proteins of these MDCK cells were labeled with biotin, lysed, solubilized, and immunoprecipitated with an anti-GFP or anti-mCherry antibody. Cell surface GFP-tagged HRG-1 was mostly detected in the fraction that contains biotinylated basolateral membrane (Figure. 6C). On the other hand, cell surface mCherry-tagged HCP1 and mCherry-tagged DMT1A-I were mostly detected in the fraction that contains biotinylated apical membrane. These results suggest that HRG-1 might transport heme through the lysosomal or the basolateral membrane, and it may not contribute to heme absorption from the diet through the apical membrane in polarized cells. Therefore, these candidates may play different roles in absorptive epithelial cells.

## Discussion

In this study, we showed the precise subcellular localizations of HOs. Previous reports on HO localizations indicated that HOs are located in the ER [22], nucleus [23], mitochondria [24], or caveola [25]. To understand heme catabolism and iron recycling in cells, it is important to determine the localizations of HOs and other molecules related to heme and iron metabolism. Heme is a prosthetic group that consists of a protoporphyrin ring and an iron atom. Because the cell membrane is not freely permeable to heme, it is necessary to allocate certain heme transporters at appropriate locations and orientations in the cells so HOs can adequately access their substrates. We constructed recombinant HOs with 2 different types of tagging molecules on their N or C termini to examine their exact localizations in cells. Before comparing their localizations with appropriate marker molecules, it is necessary to make sure that the addition of tagging molecules has no effect on HO localizations. Our results show that HO-1 and HO-2 clearly colocalized with each other without any influence from the tagging molecules.

Both HO-1 and HO-2 did not colocalize with PDI (Figure. 1-A, d, e, f), which is mainly located in the rough ER, but partly colocalized with syntaxin 17 and calnexin (Figure. 1A, g-l), both of which are located in smooth and rough ER [33,34]. NADPH-cytochrome P450 reductase, which supplies an electron to HOs and is reported to be located in smooth ER [32], showed a clear colocalization with HOs (Figure. 1B, a, b, c). Subcellular fractionation study confirmed the specific location of HOs in smooth ER, as obtained by immunofluorescence analyses (Figure. 1B). HO localizations are discussed in previous reports, and this study shows that HO-1 does not change its location even under hemin treatment.

Humans incorporate two-thirds of the total absorbed iron as heme in enterocytes and recycles iron from senescent RBCs in macrophages. Two candidate molecules are thus far reported as heme transporters [27,28]: HCP1, which is believed to transport heme [27] or folate [29] through the plasma membrane, and HRG-1, which was identified in *C. elegans *by genome-wide microarrays as a heme-regulated gene. HRG-1 is also proven to be an essential molecule for erythropoiesis and development in zebrafish, and has a heme-uptake activity in human cultured cells and *Xenopus laevis *oocytes [28]. To investigate on which organelles these 2 molecules function, we analyzed the localizations of HRG-1 and HCP1 in non-polarized epithelial cells. Both HRG-1 and HCP1 are cotransfected in MDCK cells, and we observed these 2 molecules to be localized to the plasma membrane and lysosome. HRG-1 is located almost equally in the plasma membrane and lysosome, whereas HCP1 is located mostly in the plasma membrane and slightly in the lysosome. A previous report on HRG-1 localization showed that HRG-1 is distributed in an intracellular compartment punctuated throughout the cytoplasm, with about 10% of total HRG-1 on the cell periphery [28], and another reported that HRG-1 is localized to the endosome and plasma membrane [38]. In addition, the location of HRG-1 was changed under serum-starvation conditions [38]. In our study, HRG-1 is localized to both the lysosome and plasma membrane, and we did not observe HRG-1 translocation under serum-starvation conditions. These differences possibly arise from the differences in the cell lines or in the expression constructs used. Both HRG-1 and DMT1 are localized to lysosome, and a more detailed analysis using endogenous HRG-1 and DMT1 will be needed in future works. DMT1, which functions as a non-heme iron transporter, has 4 isoforms [39]. Alternative splicing of the DMT1 gene produces 2 distinct classes of DMT1 transcripts, which differ in the C-terminal amino acids and subsequent 3'-untranslated regions. One form, IRE (I) (Figure. 4A), has an iron responsive element (IRE) by which intracellular iron concentration can affect its translation; the other form, non-IRE (II), does not have IRE on its mRNA. Alternative use of DMT1 gene promoters generates 2 variant DMT1 transcripts that differ in nucleotide sequences encoding the 5'-untranslated region and their subsequent N-terminal amino acids. A polypeptide transcribed from the 5'-upstream promoter and exon 1A is indicated as 1A, and a polypeptide transcribed from another promoter and exon 1B is indicated as 1B in Figure. 4A. These 4 DMT1 isoforms showed distinct subcellular localizations. DMT1A-I is mainly located in the plasma membrane, DMT1B-I in late endosome and lysosome, and both DMT1A-II and DMT1B-II in the recycling endosome. The protein expression levels of these 4 isoforms differ among tissue types; DMT1A-I is expressed in the duodenum and kidney and absorbs non-heme iron into the cytosol, whereas DMT1B-I is expressed in the macrophage and transports non-heme iron from the lysosome into the cytosol. DMT1A-II is expressed in the duodenum; its expression level is considerably lower compared with other isoforms. DMT1B-II is expressed in peripheral tissues and transports iron released from transferrin in the recycling endosome [40]. Notably, we investigated the crucial signal for DMT1A-I localization to the plasma membrane. DMT1A-I L16A mutant localizes to the late endosome and lysosome. We reported that the non-IRE (II) C-terminal cytosolic region conducts the proper endosomal recycling of DMT1A and DMT1B [9]. As a next step, analysis of the detailed function of N-terminal cytosolic region in the sorting mechanism of DMT1A is needed.

We compared the localization of HRG-1 with that of HCP1 in epithelial cells: HRG-1 colocalized with HCP1 in the plasma membrane in non-polarized epithelial cells. On the other hand, HRG-1 and HCP1 show different localizations in polarized epithelial cells; HCP1 is located in the apical membrane and HRG-1 is in the basolateral membrane and lysosome. HRG-1 can be detected very slightly in apical membrane in this system, and we will examine in the next stage whether endogenous HRG-1 is localized in apical membrane and can function to uptake heme from diet. HCP1 is able to transport folate more efficiently than heme [27,29,41]. HCP1 has a higher affinity for folate (Km = 1.67 μM) than heme (Km = 125 μM), and thus folate may be the more physiologically relevant target of HCP1. However, our localization study indicates that HRG-1 cannot function as a heme transporter to absorb heme from diet because of its location in epithelial cells, and that HCP1 may play a role in dietary heme uptake, because heme concentration in meat is roughly estimated to be 100 - 250 μM [42].

## Conclusions

**Figure 7:**
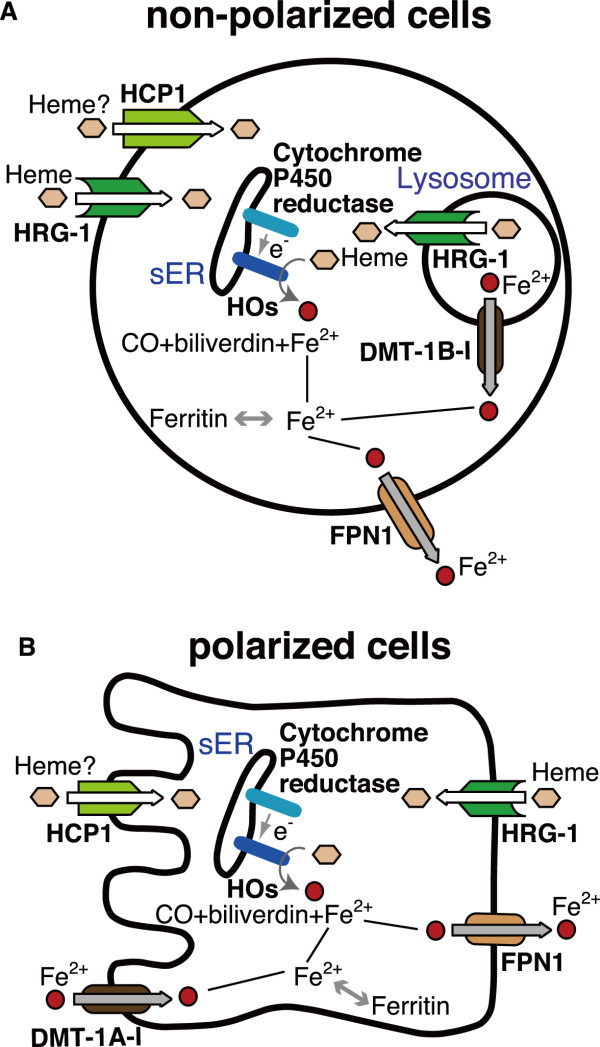
**Summary of heme and iron transport**. Schemas A and B indicate the possible mechanisms of heme transport and catabolism in non-polarized and polarized cells, respectively. See Discussion and Conclusion for further details.

We summarize a putative heme transport and iron recycling pathway in Figure. 7. In non-polarized cells (Figure. 7A), both HRG-1 and HCP1 are located in the plasma membrane and can mediate heme uptake from body fluids. HRG-1 is located in the lysosome and can transport heme from the inside of the lysosome. "Professional" phagocytic cells such as macrophages are one of the most important non-polarized cells that can scavenge senescent RBCs and the hemopexin-heme and haptoglobin-hemoglobin complexes from the bloodstream. We therefore propose that there are 2 putative heme uptake pathways present in the macrophage: one is direct uptake of heme from body fluids to the cytosol via HRG-1 or HCP1 on the plasma membrane, and the other is through the lysosomal membrane. In the latter pathway, lysosomal heme is derived from the heme complexes that are endocytosed by the CD163 hemopexin-heme [43] or LRP1/CD91 haptoglobin-hemoglobin [44] receptors, and the senescent RBCs that are phagocytosed and digested in the lysosome. Lysosomal heme is transported through HRG-1 into the cytosol. Once heme is incorporated into the cytosol, it can be degraded by the aid of HOs localized just at the cytosolic side of smooth ER. Released iron from heme is excreted into body fluids via FPN1 and reutilized as cofactors for many enzymes and proteins, such as hemoglobin and mitochondrial cytochrome. In polarized cells (Figure. 7B), HCP1 and DMT1A-I are localized to the apical membrane, and absorb heme and non-heme iron from diets, respectively. HRG-1 is located in the basolateral membrane and transport heme from body fluids into the enterocyte. The significance of this molecule as a heme transporter in enterocytes is not clear thus far.

In this study, we analyze the localizations of HOs, HRG-1, HCP1, and DMT1 in non-polarized and polarized cells, and add new knowledge concerning heme transport and iron recycling system. Future work will be needed to further define the functions of HRG-1 and HCP1 in enterocytes and macrophages, the significance of HRG-1 localization to the basolateral membrane in enterocytes, the capability of HRG-1 to transport heme from body fluids or lysosome in macrophages, and the capability of HCP1 to transport heme from diet.

## Methods

### Antibodies and reagents

Mouse anti-human transferrin receptor (TfR) monoclonal antibody (mAb) (N-2) was prepared as described previously [30]. Mouse anti-human EEA1 mAb, mouse anti-human GW130 mAb, mouse anti-human calnexin mAb, and mouse anti-human α-tubulin mAb were purchased from BD Transduction Laboratories (San Jose, CA). Mouse anti-HA mAb was purchased from Covance (Berkeley, CA), and mouse anti-human protein disulfide isomerase (PDI) mAb was purchased from Daiichi Fine Chemical (Toyama, Japan). Mouse anti-human LAMP2 mAb (H4B4, developed by Drs. J.E.K. Hildreth and J.T. August) was obtained from the Developmental Studies Hybridoma Bank (Baltimore, MD). Alexa 594-labeled anti-rabbit IgG and anti-mouse IgG, Alexa 488-labeled anti-rabbit IgG and anti-mouse IgG, and MitoTracker Deep Red 633 were purchased from Invitrogen Corp. (Carlsbad, CA).

The amino-acid-coding regions of human HOs, NADPH-cytochrome P450 reductase, syntaxin 17, HCP1, and HRG-1 were amplified using HEp-2 or Caco-2 cell cDNAs as templates. The fragments containing the full-length ORF were ligated into pEGFP-C1, pEGFP-N1, and pIRES-HA vectors (Clontech, Palo Alto, CA). pRSET-B-mCherry vector was kindly provided by Dr. Roger Y. Tsien (University of California, San Diego, CA). The mutant forms of DMT1 were made by PCR mutagenesis using KOD plus DNA polymerase (Toyobo, Osaka, Japan). Nucleotide sequences of PCR-oriented constructs were confirmed by the dideoxynucleotide chain-termination method using an ABI 3100 automated DNA sequencer.

### Cell culture and transfection

Human HEp-2 epithelial cells and Madin-Darby canine kidney (MDCK) cell line were maintained in high-glucose Dulbecco's minimal essential medium containing 10% fetal calf serum, 50 μg/ml penicillin, and 50 μg/ml streptomycin. FuGENE 6 transfection reagent (Roche Molecular Biochemicals, Mannheim, Germany) was used for the transfection of HEp-2 cells and MDCK cells, which was done according to the manufacturer's instructions. After transfection, cells were cultured for 48 h on glass coverslips. For the polarity studies and cell surface labeling assay, clonal MDCK cells were cultured as confluent monolayers on polycarbonate filter chamber (Transwell, Corning, NY) for 6 days.

### Immunofluorescence microscopy

Cells grown on glass coverslips and Transwell were fixed with 4% paraformaldehyde (PFA) in PBS for 15 min at room temperature, and permeabilized with 0.2% Triton X-100 in PBS for 20 min. The coverslips and Transwell were washed and blocked in 0.1% fish skin gelatin in PBS. Cells were incubated with primary antibodies for 60 min at room temperature. Coverslips and Transwell were washed with 0.1% fish skin gelatin in PBS. Secondary antibodies coupled to Alexa 488 or Alexa 594 were incubated on cells for 60 min at room temperature. MitoTracker Deep Red was used to stain the mitochondria. HEp-2 cells grown on coverslips were incubated in fresh medium with 250 nM MitoTracker Deep Red at 37°C for 45 min. Coverslips and Transwell were washed and mounted on slides with VECTASHIELD (Vector Laboratories, Burlingame, CA). The XY and XZ images were obtained by using a Leica TCS SP2 AOBS confocal laser scanning microscope system.

### Cell surface labeling

Cells grown on Transwell were washed with ice-cold PBS containing 0.1 mM CaCl_2 _and 1 mM MgCl_2 _[PBS(+)]. For selective labeling of the apical or the basolateral surface, sulfo-NHS-LC-biotin was added either to the apical or the basolateral compartment of the filter chamber. The compartment not receiving sulfo-NHS-LC-biotin was filled with an equivalent volume of PBS(+). Three filter chambers were used per experimental condition. Cells were washed with Tris-buffered saline with mild agitation. Then, cells were extracted in RIPA buffer [150 mM NaCl, 50 mM Tris (pH 8.0), 5 mM EDTA, 1% Nonidet P-40, 0.5% deoxycholate, and 0.1% SDS] and the extracts were clarified by centrifugation at 14,000 × *g *for 15 min. The resulting supernatants were added to anti-green fluorescent protein (GFP) or anti-mCherry antibody conjugated protein A beads. After 2-h incubation, the beads were washed and the proteins were eluted with Laemmli buffer. Eluants were analyzed by immunoblotting. Biotinylated proteins were detected by ImmunoPure Avidin, Horseradish Peroxidase, Conjugated (Pierce Biotechnology, Rockford, IL).

### Subcellular fractionation

HEp-2 cells stably expressing HA-tagged HO-2 were grown to confluency in a 100-cm^2 ^dish. Cells were washed once with PBS and scraped in homogenizing buffer (0.25 M sucrose and 10 mM Tris-HCl, pH 7.4). Cells were homogenized in glass-Teflon Potter homogenizer rotating at 1,000 rpm, 25 strokes. The homogenate was centrifuged at 1,000 × *g *for 10 min to obtain post nuclear supernatant (PNS). PNS was centrifuged at 10,000 × *g *for 20 min to obtain the post-mitochondrial fraction (PMF). Then, to obtain smooth and rough microsomal fractions, PMF was loaded onto 3.5 ml of 1.3 M sucrose. Samples were centrifuged at 100,000 × *g *for 2 h at 4°C. Aggregated rough microsomes sedimented through the 1.3 M sucrose layer and pelleted at the bottom of the tubes, whereas smooth membranes collected on top of the 1.3 M sucrose layer [31].

### Western blot analysis

HEp-2 cells were cultured with 100 μM hemin for 36 h. After incubation, cells were washed in cold PBS. Proteins were collected in tube and solubilized in 2% SDS-Laemmli buffer; protein contents were determined by the method of Lowry. The samples were resolved by SDS-PAGE using 12% acrylamide gels and blotted onto polyvinylidene difluoride membranes. HO-1 was detected by rabbit anti-HO-1 polyclonal antibody (Stressgen, Victoria, BC, Canada).

### Recombinant constructs

The recombinant constructs used in this study are indicated in Additional file 1 Figure. S1A.

## Abbreviations

DMT1: divalent metal transporter 1; GFP: green fluorescent protein; HA: human influenza hemagglutinin; HCP1: heme carrier protein 1; HO: heme oxygenase; HRG-1: heme responsive gene-1; FPN1: ferroportin 1; PBS: phosphate buffered saline; PDI: protein disulfide isomerase; PFA: paraformaldehyde; TfR: transferrin receptor.

## Authors' contributions

IY designed and implemented all localization experiments, prepared all final figures, and drafted this article. MT performed the cell surface labeling assay. YK constructed the fusion protein plasmids. YY performed the subcellular fractionation. RA participated in developing the methods for localization experiments. FK conducted this study, supervised the results, and finalized the manuscript. All authors read and approved the final manuscript.

## Supplementary Material

Additional file 1 **Figure S1: Recombinant constructs**. A. Schematic representation of recombinant HOs, NADPH-cytochrome P450 reductase, HRG-1, HCP1, DMT1, and syntaxin 17 showing the locations of transmembrane (TM) domains (blue box) and the order of tags. TM domains were predicted using the TMHMM program. HO-1 and HO-2 have a single TM domain at the C-terminal region, and NADPH-cytochrome P450 reductase has a single TM domain at the N-terminal region. HRG-1, HCP1, DMT1, and syntaxin 17 have multiple TM domains. These proteins are tagged with HA, GFP, or mCherry as depicted. B. GFP-tagged HO-1 and HA-tagged HO-1 were cotransfected in HEp-2 cells and visualized by confocal microscopy. Recombinant construct combinations were N-terminally GFP-tagged HO-1 (a and g), C-terminally GFP-tagged HO-1 (d and j), N-terminally HA-tagged HO-1 (b and k), and C-terminally HA-tagged HO-1 (e and h). C. GFP-tagged HO-1 and HA-tagged HO-2 were cotransfected into HEp-2 cells and stained with antibody against GFP (a) and HA (b). Each inset shows a higher magnification image of the boxed area. Click here for file

Additional file 2 **Figure S2: Subcellular localizations of HOs**. GFP-tagged HO-1 was transfected into HEp-2 cells and visualized by confocal microscopy. The cells were fixed and incubated with antibodies against GFP (a, d, g, and j), TfR (b), LAMP2 (e), EEA1 (h), and caveolin (k). Click here for file

Additional file 3 **Figure S3: HRG-1 does not change its location under starvation conditions**. HEp-2 cells stably expressing GFP-tagged HRG-1 were cultured in complete medium (DMEM + 10% FBS) (a), or in starvation medium (excluding FBS) for 2 h (b) or 24 h (c). MDCK cells stably expressing GFP-tagged HRG-1 were cultured on Transwell for 6 days after confluence. Cells were cultured in complete medium (DMEM + 10% FBS) (d), or in starvation medium (excluding FBS) for 2 h (e) or 24 h (f). Click here for file
