# Supplementary figures and images for: Heme and non-heme iron transporters in non-polarized and polarized cells
Source: BMC Cell Biol. 2010 Jun 4;11:39. doi: 10.1186/1471-2121-11-39 (PMC3224662; doi:10.1186/1471-2121-11-39)

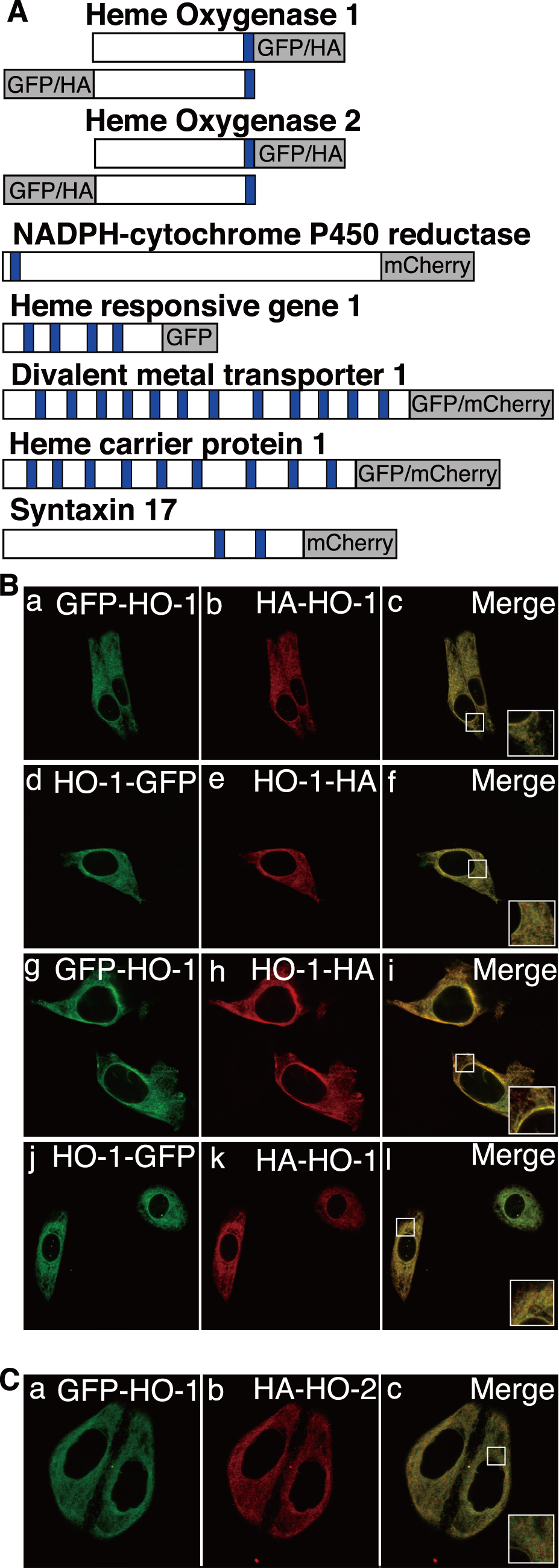

Supplement: Additional file 1 — Figure S1: Recombinant constructs. A. Schematic representation of recombinant HOs, NADPH-cytochrome P450 reductase, HRG-1, HCP1, DMT1, and syntaxin 17 showing the locations of transmembrane (TM) domains (blue box) and the order of tags. TM domains were predicted using the TMHMM program. HO-1 and HO-2 have a single TM domain at the C-terminal region, and NADPH-cytochrome P450 reductase has a single TM domain at the N-terminal region. HRG-1, HCP1, DMT1, and syntaxin 17 have multiple TM domains. These proteins are tagged with HA, GFP, or mCherry as depicted. B. GFP-tagged HO-1 and HA-tagged HO-1 were cotransfected in HEp-2 cells and visualized by confocal microscopy. Recombinant construct combinations were N-terminally GFP-tagged HO-1 (a and g), C-terminally GFP-tagged HO-1 (d and j), N-terminally HA-tagged HO-1 (b and k), and C-terminally HA-tagged HO-1 (e and h). C. GFP-tagged HO-1 and HA-tagged HO-2 were cotransfected into HEp-2 cells and stained with antibody against GFP (a) and HA (b). Each inset shows a higher magnification image of the boxed area. [file 1471-2121-11-39-S1.TIFF]

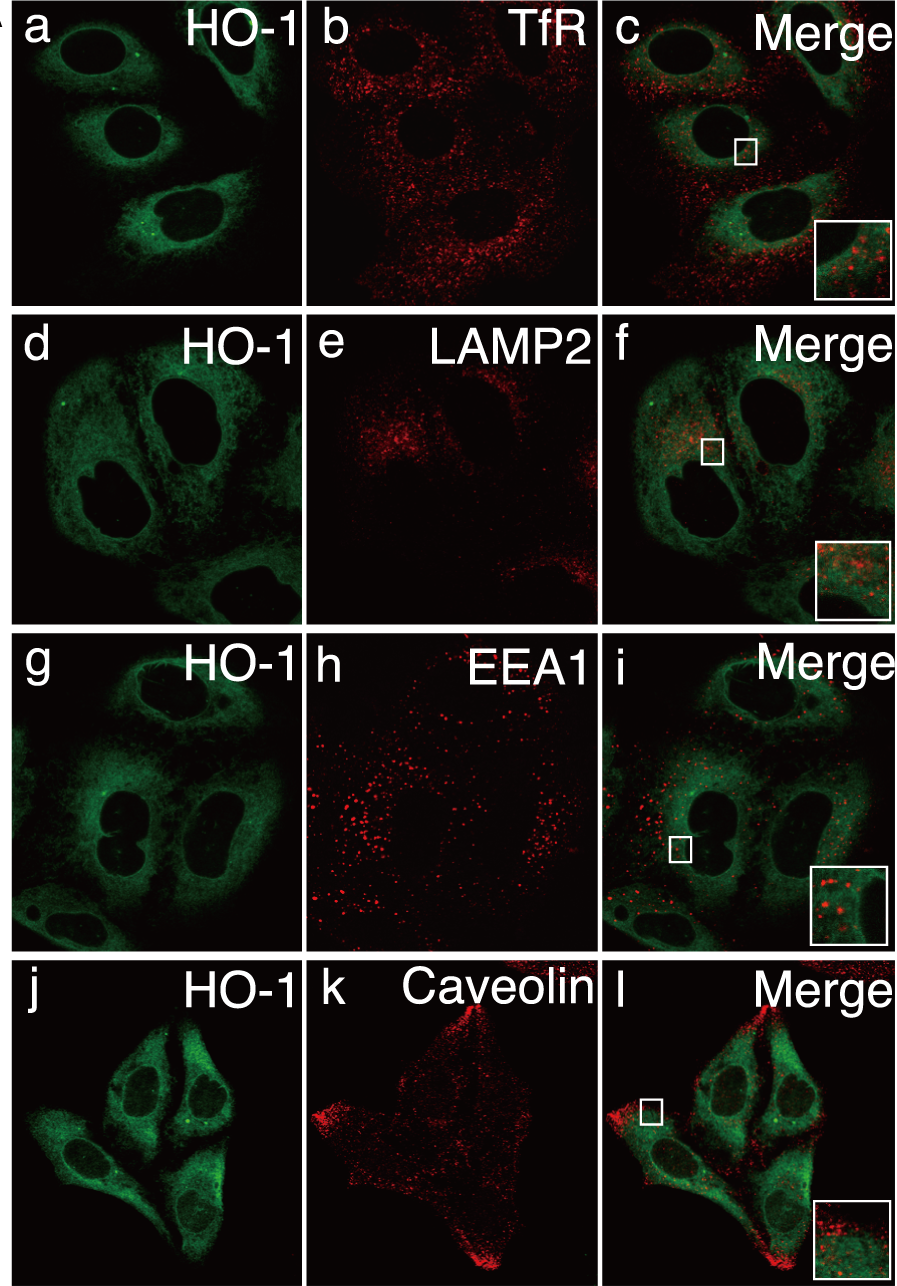

Supplement: Additional file 2 — Figure S2: Subcellular localizations of HOs. GFP-tagged HO-1 was transfected into HEp-2 cells and visualized by confocal microscopy. The cells were fixed and incubated with antibodies against GFP (a, d, g, and j), TfR (b), LAMP2 (e), EEA1 (h), and caveolin (k). [file 1471-2121-11-39-S2.TIFF]

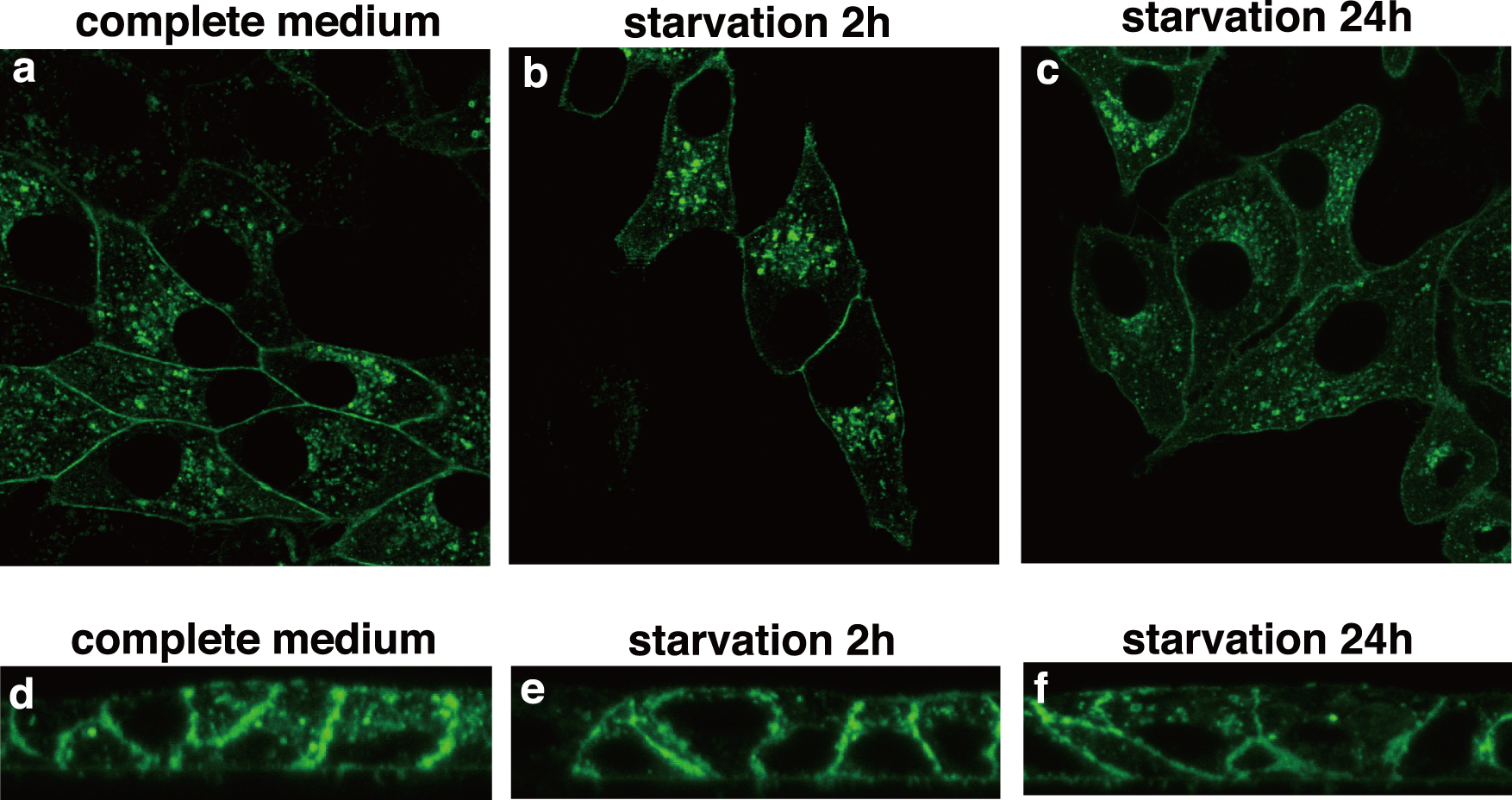

Supplement: Additional file 3 — Figure S3: HRG-1 does not change its location under starvation conditions. HEp-2 cells stably expressing GFP-tagged HRG-1 were cultured in complete medium (DMEM + 10% FBS) (a), or in starvation medium (excluding FBS) for 2 h (b) or 24 h (c). MDCK cells stably expressing GFP-tagged HRG-1 were cultured on Transwell for 6 days after confluence. Cells were cultured in complete medium (DMEM + 10% FBS) (d), or in starvation medium (excluding FBS) for 2 h (e) or 24 h (f). [file 1471-2121-11-39-S3.TIFF]
